# Verantwortung für Frieden, Freiheit und Wohlstand – außen- und sicherheitspolitische Grundsätze der CDU/CSU

**DOI:** 10.1007/s12399-021-00848-7

**Published:** 2021-05-14

**Authors:** Johann David Wadephul

**Affiliations:** Fraktion der CDU/CSU im Deutschen Bundestag, Platz der Republik 1, 11011 Berlin, Deutschland

**Keywords:** Wertegebundene Außenpolitik, Europa, Transatlantische Beziehungen, NATO, Freihandel, Multilateralismus, Regel- und wertebasierte Ordnung, Value-based foreign policy, Europe, Transatlantic relationship, NATO, Free trade, Multilateralism, Rules- and value-based international order

## Abstract

Um die stark unter Druck geratene regel- und wertebasierte Ordnung der Welt besser zu schützen, muss sich Deutschland, die viertgrößte Wirtschaftsmacht der Welt, in der Außen- und Sicherheitspolitik stärker einbringen, seine Interessen klarer formulieren und konsequenter verfolgen. Die CDU/CSU folgt dabei einem klaren und bewährten Wertekompass: Wir bekennen uns zur EU, NATO, den Vereinten Nationen und zu multilateralem Handeln. Wir gestalten die Welt gemeinsam mit den demokratischen Rechtsstaaten der Welt, die unsere Wertepartner sind, schaffen und vertiefen Netzwerke und bauen gemeinsam Resilienzen auf.

## Abstract (en)

The rules- and value-based international order is under strain: Germany, as the fourth-largest economy in the world, has to develop a more active foreign and security policy. Germany should define and pursue its own interests more clearly. In doing so, the CDU/CSU is guided by clear and well-established values: We are deeply committed to the EU, NATO, the UN and multilateral action. Together with democratic countries who are governed by the rule of law and who share our values, we endeavour to shape the international order, bolster our international networks, and cooperate to collectively build our resilience.

## Einleitung

Ein Blick auf die außen- und sicherheitspolitische Lage weltweit, wie sie sich dem Betrachter^1^ zu Beginn des Jahres 2021 bietet, ist nicht nur ernüchternd – er ist alarmierend. Lang andauernde Krisen und Konflikte schwelen weiter: angefangen von Syrien über die Golfregion bis hin zur Sahelzone im Westen Afrikas. Neue Konflikte sind mit großer Vehemenz ausgebrochen: so etwa im Südkaukasus und in Äthiopien. Spannungen nehmen in vielen Regionen zu und wir stehen vor einer ganzen Reihe Kalter Kriege. Umbrüche und geopolitische Verschiebungen bestimmen die langfristige Tendenz. Die regel- und wertebasierte Ordnung der Welt, wie sie seit über 70 Jahren trotz aller politischen und institutionellen Schwächen für die Staatengemeinschaft Stabilität und immer wieder weitgehend Frieden gebracht hat, erodiert seit Jahren mehr und mehr. Verschiedene Akteure versuchen aktiv, die Regeln des friedlichen Miteinanders der Völker, völkerrechtliche Standards etwa im Bereich des Seerechts oder der Menschenrechte, den offenen und faktenbasierten öffentlichen Diskurs in anderen Ländern oder gar die staatliche Integrität und Souveränität fremder Staaten sowie die internationalen Institutionen zu ihrer Wahrung zu unterminieren oder zu missachten. Zudem hat die Covid-19-Pandemie im vergangenen Jahr auch in der Außen- und Sicherheitspolitik viele bestehende Entwicklungen im Guten wie im Schlechten beschleunigt und teilweise auch ganz neue Entwicklungen in Gang gesetzt.

Deutschland ist in Europa mit Abstand die größte, weltweit gesehen die viertgrößte Wirtschaftsmacht – nur die USA, China und Japan haben ein noch größeres Bruttoinlandsprodukt. Als Exportnation hat unser Land ein besonderes Interesse an einer dauerhaft friedlichen, gerechten und damit stabilen Ordnung in Europa und der Welt. Gegenüber Störungen und Destabilisierungen der internationalen Ordnung ist unser Land vor allem aufgrund seiner weltweiten engen wirtschaftlichen Verbindungen besonders verletzlich. Umso mehr muss sich Deutschland in der Außen- und Sicherheitspolitik stärker einbringen, seine Interessen klarer formulieren und konsequenter verfolgen. Auch unsere europäischen Partner erwarten, dass Deutschland, bei aller Offenheit zum Kompromiss, mit klarem sicherheitspolitischem Kompass agiert. Nach innen hat die deutsche Politik die Aufgabe, den Bürgern zu erklären und mit den Bürgern zu diskutieren, aus welchen Gründen und in welchen Zusammenhängen deutsche Interessen formuliert und verfolgt werden.

Deutschland muss seine Interessen klarer als bisher definieren, besser nach außen kommunizieren und konsequent im Sinne seiner Interessen handeln – nur so wird Deutschland seinen Einfluss sichern und stärken können. Dies gilt für diplomatische Initiativen, entwicklungspolitische Beiträge und das Einbringen militärischer Fähigkeiten. Mehr noch als in der Vergangenheit müssen wir bereit sein, zusammen mit unseren Verbündeten und Partnern und unter Wahrung der völkerrechtlichen und unserer verfassungsrechtlichen Vorgaben auch militärische Mittel anzuwenden.

Die Bürger unseres Landes und Europas haben einen Anspruch auf eine leistungsfähige und glaubwürdige Sicherheitsvorsorge. Die CDU/CSU ist Garant dafür, dass diese vornehmste und existenzielle Aufgabe des Staates nicht zu Lasten kommender Generationen vernachlässigt wird: Deutschlands Investition in Sicherheit ist die Grundlage für den Erhalt von Frieden, Freiheit und Wohlstand für unser Land und Europa auch in Zukunft.

## Deutsche Außen- und Sicherheitspolitik in multilateralen Netzwerken

Bei alledem handeln wir nicht allein. Wir stehen für multilaterale Zusammenarbeit zur Bewältigung globaler Herausforderungen.

Deutschland und andere offene, liberale Demokratien geraten immer stärker in den Interessensgegensatz zu autoritären, staatskapitalistischen Regimen. Es ist im deutschen und europäischen Kerninteresse, diese Systemkonkurrenz in unserem Sinne zu bestehen. Durch die Verteidigung politischer und persönlicher Freiheiten werden wir immer einen Attraktivitätsvorsprung gegenüber autoritären Herrschaftsformen erhalten. Deswegen kommt unserem Außenhandeln und unserer Verteidigungspolitik eine existenzielle Bedeutung zu: Sie sichern unser Gesellschafts- und Wirtschaftsmodell und unsere Werte. Die Zusammenarbeit in der Europäischen Union, mit unseren transatlantischen Partnern und mit unseren Wertepartnern weltweit muss zu einer durchsetzungsfähigen Interessensgemeinschaft weiterentwickelt werden.

### Das transatlantische Bündnis als Kern unserer Außen- und Sicherheitspolitik

Die Wahl von Joe Biden als Präsident der Vereinigten Staaten bedeutet eine große Chance: Wir haben in den letzten vier Jahren mit zunehmender Fassungslosigkeit mit ansehen müssen, wie die amerikanische Regierung die regelbasierte internationale Ordnung, die sie einst selbst geschaffen hat, Schritt für Schritt infrage stellte und schwächte – als Beispiele seien hier nur das Klimaabkommen, das Atomabkommen mit dem Iran (Joint Comprehensive Plan of Action, JCPOA), die Weltgesundheitsorganisation (WHO) und die Welthandelsorganisation (WTO) genannt. Die Besetzung des Kapitols in der amerikanischen Hauptstadt durch gewaltbereite Demonstranten hat gezeigt, wie verletzlich die innenpolitische Situation in den USA und wie tief die Spaltung in dem Land ist. Ein neuer US-Präsident Biden wird darum absehbar große Teile seiner Energie für die Abmilderung der Spaltung in seinem Land aufwenden müssen. Dennoch haben wir auch die begründete Hoffnung, in der neuen US-Regierung in der Zukunft einen Partner zu haben, dem wieder verstärkt an internationaler Verantwortungsübernahme gelegen ist. Umso wichtiger ist es für uns als Deutsche und als Europäer, diese Gelegenheit zu nutzen: Wir sollten uns jetzt als verlässlicher Partner im transatlantischen Bündnis zeigen, der willens ist, auf Augenhöhe mit den USA international zusammenzuarbeiten. Doch deswegen müssen wir selbst viel tun: Wir müssen das 2014 in Wales durch die Bundesregierung vereinbarte und seitdem wiederholt bekräftigte Ziel, 2 % des Bruttoinlandsprodukts in Verteidigungsausgaben zu investieren, zügig umsetzen und uns als handlungsfähiger und -williger Akteur erweisen, indem wir neue Initiativen zur Stärkung des transatlantischen Bündnisses und neue transatlantische Initiativen zur Lösung und Entschärfung sicherheitspolitischer Probleme in der Welt auf den Weg bringen.

Die NATO ist und bleibt das Rückgrat der euroatlantischen Sicherheit. Auch für die Außen- und Sicherheitspolitik der CDU/CSU bleibt die transatlantische Partnerschaft Kern und Hauptbezugspunkt. Die NATO muss wieder zentrales Dialogforum sein, um gemeinsam Lösungen für die großen Herausforderungen unserer Zeit zu finden. Darum müssen wir den Reflexionsprozess im Bündnis nutzen, um den politischen Kern der NATO zu stärken, und das Bündnis mit einem neuen Strategischen Konzept über ein Jahrzehnt nach der Erstellung des vorangegangenen auf die Herausforderungen von heute einstellen. Gemeinsam mit unseren europäischen Partnern müssen wir stärker als bisher für Stabilität in unserer Nachbarschaft und unsere sicherheitspolitischen Interessen in der Welt Sorge tragen. Darum müssen wir den europäischen Pfeiler der NATO stärken, im Bündnis mehr Verantwortung übernehmen und für eine faire Lastenverteilung eintreten. Deutschland muss in der Mitte unserer Partner mit überzeugendem Beispiel vorangehen. Für uns gilt der Leitsatz: Transatlantisch bleiben, europäischer werden.

### Europa handlungsfähig machen

Kern unserer Bemühungen muss es sein, die EU auch in Fragen der Außen- und Sicherheitspolitik zu einem berechenbaren und handlungsfähigen Akteur zu machen. Dafür müssen wir die Europäische Verteidigungsunion hin zu einer Armee der Europäer ausbauen und dafür sorgen, dass zum einen die bestehenden Mittel effizienter genutzt werden. Zum anderen aber müssen wir auch gemeinsam neue Fähigkeiten komplementär zur NATO aufbauen, um effektiver zu werden.

Wir wollen die Handlungsfähigkeit der EU durch das qualifizierte Mehrheitsverfahren in der Außen‑, Sicherheits- und Verteidigungspolitik stärken. Wir sprechen uns in diesem Zuge dafür aus, einen Europäischen Sicherheitsrat einzurichten. Er soll eine schnellere Reaktion der EU in der Außenpolitik ermöglichen, die EU-Außenpolitik klarer und eindeutiger formulieren und er soll sicherstellen, dass diese durch seine Mitglieder als Repräsentanten der gesamten EU gemeinsam und verbindlich vertreten und umgesetzt wird. Die Zusammenarbeit mit Großbritannien wird auch nach dem Austritt des Vereinigten Königreichs aus der EU von zentraler Bedeutung sein.

### Die Vereinten Nationen als Herzstück der regelbasierten internationalen Ordnung

Die Vereinten Nationen sind das Herzstück der nach dem Zweiten Weltkrieg aufgebauten internationalen Ordnung, die auf allgemeinverbindlichen Regeln für alle und auf zentralen Wertentscheidungen wie dem Respekt der Menschenrechte und der Rechtstaatlichkeit basiert. Gleichwohl müssen die UN zur Regelung internationaler Herausforderungen entscheidungs- und handlungsfähiger werden. Es gilt gegen wachsende Bestrebungen anzugehen, diese regelbasierte Ordnung auszuhebeln, um dem Auftrag der UN entsprechend Frieden, Menschenrechten, Freiheit, Demokratie und Rechtsstaatlichkeit zum weltweiten Durchbruch zu verhelfen und zur Bewahrung der Schöpfung und zur Weiterentwicklung des Völkerrechts beizutragen. Für diese Ziele müssen die weltweiten Wertepartner gemeinsam wirksamer eintreten.

Zudem sollten wir klar adressieren, welche Akteure die UN und die regelbasierte internationale Ordnung zu schwächen und auszuhöhlen suchen: Die beiden Veto-Mächte Russland und China blockieren den Sicherheitsrat immer wieder in entscheidenden Momenten. Seien es die Kriege in Syrien und Libyen oder das iranische Atomwaffenprogramm – immer sind es diese beiden Vetomächte, die echte multilaterale Lösungen im UN-Rahmen verhindern

Gleichzeitig stellen wir fest: Wenn es um die Stärkung der Handlungsfähigkeit und Wirksamkeit der UN geht, richten sich die Augen ganz automatisch auch auf Deutschland. Deswegen beanspruchen wir auch für uns einen Ständigen Sitz im UN-Sicherheitsrat, gemeinsam mit unseren G4 Partnerländern Japan, Indien und Brasilien. Der Sicherheitsrat der UN spiegelt in seiner derzeitigen Zusammensetzung immer noch die Welt von vor 75 Jahren wider. Nur durch eine Anpassung auch dieses zentralen Gremiums an die globalen Kräfteverhältnisse können wir die Glaubwürdigkeit der UN weiter stärken. Dabei ist klar: Die Entscheidungsfähigkeit des Sicherheitsrates darf durch weitere Veto-Rechte nicht noch weiter reduziert werden.

## Nachbarn, Partner, Herausforderungen und Krisenregionen

### Europas Nachbarschaft

Die EU braucht eine möglichst enge Zusammenarbeit mit den Staaten ihrer unmittelbaren Nachbarschaft, um sich gegen die wachsenden Herausforderungen der Globalisierung und den zunehmenden Zerfall der internationalen Ordnung besser behaupten zu können. Die Politik der Erweiterung und der Heranführung von europäischen Staaten an die EU muss Europas Sicherheit sowie sein Gewicht und seinen Einfluss in der Welt stärken.

Vor künftigen Erweiterungen müssen die Handlungsfähigkeit und die Geschlossenheit der EU deutlich verbessert werden. Die Herstellung der Erweiterungsfähigkeit ist Voraussetzung für die Aufnahme neuer Mitglieder, die für ihre Annäherung wie auch für ihre Mitgliedschaft die dafür vorgesehenen Kriterien vollständig erfüllen müssen. Der europäische Erweiterungsprozess muss zur Stärkung der europäischen Identität beitragen. Insofern wird die Vollmitgliedschaft in der Europäischen Union nicht in jedem Fall die einzige Perspektive sein. Als Zwischenschritte oder auch als dauerhafte Lösungen für die Anbindung an die EU können Assoziierungsverträge, besondere Partnerschaften, eine erweiterte Zollunion, multilaterale Kooperationsmodelle wie der Europäische Wirtschaftsraum oder Teilmitgliedschaften vereinbart werden.

Unser Ziel ist es, durch enge, stabile und nachhaltige Nachbarschaftsbeziehungen auf der Grundlage der gemeinsamen Werte des Europarates die Unabhängigkeit unserer östlichen Partner zu stärken sowie ihre politische und wirtschaftliche Modernisierung zu fördern. Wir setzen uns dafür ein, dass der europäische Weg der Ukraine zu einem modernen demokratischen, rechtstaatlichen und wirtschaftlich starken engen Partner von EU und NATO eine Erfolgsgeschichte wird. Die Zukunft Europas entscheidet sich auch in der Ukraine.

### Die Beziehungen zu Russland verbessern

Wir streben gute Beziehungen und eine enge Zusammenarbeit mit Russland an. Zunächst einmal müssen wir die weitgehende Sprachlosigkeit zwischen Russland und der EU überwinden. Wir wollen schrittweise mehr Vertrauen aufbauen, um zu einer an gemeinsamen Werten und Regeln orientierten Sicherheitsarchitektur in Europa zurückzukehren. In der Vergangenheit mussten wir jedoch feststellen, dass Russland seinen Nachbarstaaten in eklatant völkerrechtswidriger Weise nur eine eingeschränkte Souveränität zubilligen möchte, um für sich eine *Einflusssphäre* zu sichern. Russland schreckt dabei auch vor dem Einsatz militärischer Gewalt (durch eigene Soldaten oder durch direkt oder indirekt gesteuerte Söldnertruppen) und vor völkerrechtwidriger Annexion nicht zurück und nimmt dabei, wie zahlreiche erschreckende Fälle in Syrien zeigen, keinerlei Rücksicht auf wehrlose Zivilbevölkerungen in Krisengebieten. Russland setzt zudem auf staatlich gesteuerte Desinformationskampagnen und massive Cyberattacken. So wurden auch in Deutschland wiederholt hohe und höchste Bundesbehörden und der Bundestag Opfer gezielter Hackerangriffe, die sich eindeutig nach Russland zurückverfolgen ließen. Des Weiteren werden Regimekritiker und Oppositionelle verfolgt und Ziele von Mordanschlägen mit Hilfe international geächteter chemischer Kampfstoffe.

EU und NATO müssen einer destabilisierenden und völkerrechtswidrigen Politik Russlands mit Geschlossenheit, Wachsamkeit, Resilienz, Standfestigkeit sowie Gestaltungswillen auch weiterhin entgegentreten und ihre politische Strahlkraft stärken. Im Sinne der Ziele des Europarates und auf der Grundlage seiner universellen Werte sind wir bereit, demokratische Entwicklungen, Rechtsstaatlichkeit, Medienfreiheit und wirtschaftliche Entwicklung in Russland zu fördern. Solange Russland anders sein will als die anderen europäischen Völker und gegen den Westen agiert, braucht das viel strategische Geduld.

### Die Türkei als wichtiger Partner

Die Türkei ist für uns ein wichtiger Partner. Unsere Beziehungen haben ein großes Potenzial. An der Schnittstelle zwischen Europa, Asien und dem Mittleren Osten ist die Türkei für die EU und die NATO von wachsender geostrategischer Bedeutung. Die Türkei muss aber ein zuverlässiger NATO-Partner bleiben. Die zunehmende Aggressivität und Rücksichtslosigkeit, die die türkische Führung bei der Verfolgung ihrer Interessen an den Tag legt, sind Indikatoren für und eine schrittweise Entfremdung von den Grundprinzipien der westlichen Wertegemeinschaft. Praktiken wie der kaum verdeckte Einsatz islamistischer Terrorkämpfer zur Durchsetzung vermeintlich höherrangiger eigener Interessen sind inakzeptabel. Eine möglichst enge Anbindung der Türkei an die EU, für die wir uns einsetzen, wird davon abhängen, wie weit die Türkei die westlichen Werte lebt, zu denen wir uns gemeinsam verpflichtet haben – insbesondere auch in den Bereichen Rechtsstaatlichkeit und Menschenrechte.

### Verantwortung für Frieden im Nahen Osten

Frieden im Nahen und Mittleren Osten liegt in unserem ureigenen Interesse. Darum müssen wir im europäischen Verbund Verantwortung für Stabilität und Sicherheit in der Region übernehmen. Deutschland und die EU müssen angesichts erkennbarer Krisen frühzeitig mittels Prävention, Mediation und politischen Verhandlungslösungen aktiv werden, um kriegerische Auseinandersetzungen zu verhindern. Zugleich unterstützen wir die Staaten der Region im Kampf gegen den Terrorismus.

Aus unserer historischen Verantwortung wächst der Wille zu einer festen und umfassenden Partnerschaft mit Israel. Wir bekennen uns zu der besonderen Verantwortung Deutschlands gegenüber Israel als jüdischem und demokratischem Staat und dessen Sicherheit. Das Existenzrecht Israels ist für uns unumstößlich und Teil der deutschen Staatsräson. Israel muss frei von Angst, Terror und Gewalt leben können. Darum müssen wir bereit sein, für die Sicherheit Israels zusammen mit weiteren Partnern einzustehen. Die jüngste Aufnahme diplomatischer Beziehungen mehrerer arabischer Staaten mit Israel begrüßen wir ausdrücklich. Darüber hinaus werden wir alles unterstützen, was ein friedliches Zusammenleben von Israelis und Palästinensern in der Region fördert und eine Zweistaatenlösung ermöglicht.

Die regionalen Aktivitäten des Irans, sein ballistisches Raketenprogramm, seine nuklearen Ambitionen und seine fortgesetzte Weigerung, das Existenzrecht Israels anzuerkennen, stehen der Sicherheit und Stabilität des Nahen und Mittleren Ostens entgegen. Wir arbeiten mit unseren Partnern weiter daran, dass Iran sein Verhalten ändert.

### Das 21. Jahrhundert wird ein asiatisches Jahrhundert

Das 21. Jahrhundert wird wesentlich von den Ländern Asiens geprägt werden. Wir setzen uns für eine immer engere und vertiefte Zusammenarbeit in verbindlichen Partnerschaften mit diesen Ländern ein. Diejenigen Länder Asiens, die als pluralistische und liberale Demokratien für Rechtsstaatlichkeit und die Stärkung der regelbasierten internationalen Ordnung eintreten, sind unsere natürlichen Partner. Dies gilt insbesondere für Japan, Indien und Südkorea. Es liegt im deutschen wie europäischen Interesse, dass unser Land bei der Gestaltung dieser Beziehungen seiner außen- und sicherheitspolitischen Verantwortung im Raum Asien-Pazifik stärker gerecht wird.

Wir haben ein starkes Interesse an guten Beziehungen zu China auf der Grundlage allgemeinverbindlicher völkerrechtlicher Regeln und Standards. Wo immer möglich, wollen wir eine enge Zusammenarbeit zur Bewältigung globaler Herausforderungen. Eine echte Partnerschaft ist jedoch nur im Rahmen eines fairen Wettbewerbs unter für beide Seiten gleichen Bedingungen möglich; dabei muss Deutschland ebenso wie Europa Herr über seine eigenen Schlüsseltechnologien und Daten sein. China will vorherrschende Weltmacht werden, stellt als Systemrivale die regelbasierte internationale Ordnung infrage und bietet für autoritäre Staaten ein vermeintlich attraktives Entwicklungsmodell. China bekennt sich in Worten zwar zur multilateralen Ordnung, höhlt diese de facto aber immer wieder aus und schwächt diese Weltordnung. Der von China langfristig und in aller Entschlossenheit geplante Umbau der Weltordnung stellt damit für uns eine echte Herausforderung dar.

China versucht, die Werte und Regeln der UN umzudefinieren – beispielsweise den Demokratiebegriff neu zu definieren und an soziale Rechte zu binden, die wichtiger als der Rechtsstaat seien, oder die Souveränität des Staates über das Völkerrecht zu setzen. Eklatante Menschenrechtsverletzungen sind nach Chinas Auffassung zulässig, wenn sie als *innere Angelegenheiten* etikettiert werden – damit lehnt China die Universalität der Menschenrechte als angebliches politisches Konzept des Westens ab, obwohl China das Regelwerk der Menschenrechtscharta mit aufgebaut hat. Die Erfahrungen der letzten Jahre veranschaulichen, in welcher Form China die bestehende werte- und regelbasierte internationale Ordnung schrittweise in eine sinozentrische Weltordnung verändern will. Worauf das hinauslaufen könnte, müssen wir täglich in Hongkong miterleben: International gültige Regeln und Vereinbarungen, die China 1984 in der gemeinsamen Erklärung mit Großbritannien festgeschrieben hat, werden missachtet und gebrochen.

Selbst chinesische Diplomaten legen inzwischen, darin unterstützt und gezielt von ihrer Regierung angewiesen, als sogenannte Wolfskrieger, eine hohe verbale (und teilweise sogar physische) Aggressivität an den Tag, wenn es um die Vertretung vermeintlich berechtigter Interessen geht. Ein trauriger Höhepunkt bisher war die offene Diffamierung Australiens mit einer Fotomontage durch einen stellvertretenden Generaldirektor des chinesischen Außenministeriums Ende November 2020. Wir müssen zur Kenntnis nehmen, dass wir in den vergangenen Jahrzehnten im Umgang mit China falsche Anreize gesetzt haben, indem wir zunehmender chinesischer Aggression nicht entschlossen genug entgegentraten. Wir werden darum stärker als bisher zusammen mit unseren transatlantischen und weltweiten Wertepartnern aufbauend auf unseren Stärken als freiheitliche demokratische Staatengemeinschaft politische, wirtschaftliche und sicherheitspolitische Resilienz aufbauen.

### Das Schicksal Afrikas hat unmittelbare Auswirkungen auf Europa

Deshalb liegt eine friedliche, stabile, wirtschaftliche und ökologische Entwicklung des Kontinents im deutschen und europäischen Interesse – nicht zuletzt, um mittel- und langfristig den Migrationsdruck zu mindern. Zu besseren Perspektiven für die Menschen und ihre Familien in Afrika beizutragen, ist für uns nicht nur eine humanitäre, sondern auch eine wirtschaftliche und geopolitische Herausforderung.

Wir unterstützen die dortigen Staaten in ihren Reformbemühungen für wirtschaftliches Wachstum, Korruptionsbekämpfung und Rechtsstaatlichkeit. Wir können aber nur unterstützend wirken. Es bedarf der Überzeugung und Eigenanstrengung der Partner in den Ländern. Ohne diese kann keine Entwicklung erfolgen. Nachhaltige wirtschaftliche Entwicklung in Afrika ist ohne Investitionen privater Unternehmen nicht möglich. Dementsprechend wollen wir dazu beitragen, Investitionsbedingungen zu verbessern, die Produktivität zu erhöhen sowie die Chancen der Digitalisierung zu nutzen.

Wir werden auch in Zukunft die am wenigsten entwickelten Länder dabei unterstützen, Armut zu reduzieren und Zugang zu staatlicher Grundversorgung wie Bildung, Wasser, Ernährung und Gesundheitssystemen zu schaffen. Unser Ziel ist es, den Ärmsten der Armen Perspektiven zu geben. Auch die Wechselbeziehung von Mensch, Tier und Umwelt – der One-Health-Ansatz – muss wichtiger Teil globaler Gesundheitspolitik sein.

Die Afrikanische Kontinentale Freihandelszone aller Mitgliedsstaaten der Afrikanischen Union (AfCFTA) bietet die Chance einer vertieften Handelspartnerschaft ganz Afrikas mit der Europäischen Union. Wir befürworten eine Vereinbarung zwischen Afrika und der EU über einen neuen Handelsrahmen, der neben der Zusammenarbeit in den Bereichen guter Regierungsführung, Frieden und Sicherheit auch den Klimaschutz und die Kooperation in Zukunftstechnologien umfasst.

In der Sahelregion müssen wir die Länder in ihren Bestrebungen unterstützen, den Terrorismus zu bekämpfen und ihre Sicherheit und Entwicklung selbst gewährleisten zu können. Hierfür bedarf es einer gemeinsamen Anstrengung Europas.

## Globale Herausforderungen, die wir als Menschheit gemeinsam bestehen müssen

Jenseits konkreter Auseinandersetzungen mit einzelnen Akteuren stehen globale Herausforderungen, die wir als Menschheit gemeinsam bestehen müssen.

### Das Erreichen der Klimaziele ist die Überlebensfrage der Menschheit

Der nachdrückliche Einsatz für diese Ziele und gegen klimaschädigendes Verhalten muss ständiger Schwerpunkt deutscher Klima-Außenpolitik sein. Hier geht es um mehr als die jeweiligen multilateralen Verhandlungen und Abkommen, es geht um das Verständnis, dass es ein globales Thema mit sehr lokalen Auswirkungen ist. Die mit dem Klimawandel einhergehenden geostrategischen Veränderungen müssen von Deutschland und der EU zusammen mit Partnern mitgestaltet werden, um unsere Interessen zu wahren. Wo der Klimawandel Frieden und Sicherheit gefährdet, muss die internationale Gemeinschaft ansetzen, bevor Konflikte ausbrechen oder eskalieren. Dafür müssen die Handlungs‑, Präventions- und Schlichtungsmöglichkeiten insbesondere der UN verstärkt werden.

### Non-Proliferation als entscheidender Beitrag zur Zukunftssicherung unseres Planeten

Je mehr Staaten weltweit über Kernwaffen verfügen, die unseren gesamten Planeten in kürzester Zeit unbewohnbar machen können, umso stärker sind wir als Menschheit insgesamt gefährdet. Da eine Welt ohne Atomwaffen auf absehbare Zeit nicht möglich sein wird, drängen wir auf eine neue Dynamik für Rüstungskontrolle und Abrüstung bei nuklearen und konventionellen Waffen weltweit. Wir werden deshalb weiter auf die Einhaltung bestehender Abkommen drängen und unterstützen neue Initiativen, die zu mehr Sicherheit beitragen. Hierzu zählen insbesondere Maßnahmen gegen die Weiterverbreitung von Massenvernichtungswaffen und ihrer Trägertechnologien.

### Menschenrechtspolitik als integraler Bestandteil unserer Außenpolitik

Die Allgemeine Erklärung der Menschenrechte und die darin verankerten universellen Rechte für alle Menschen sind für uns der größte historische Fortschritt, der global jemals für die Würde der Menschen erreicht werden konnte. Der Schutz der Menschenwürde und die Achtung der Menschenrechte sind deshalb integraler Bestandteil unserer internationalen Politik. Wir werden weiterhin mit unseren Wertepartnern für eine Weltordnung arbeiten, in der Menschenrechte und Grundfreiheiten uneingeschränkt gelten.

Wenn autoritäre Regime Menschenrechtsverletzungen als angeblich *innere Angelegenheiten* bezeichnen und auf Nichteinmischung pochen, bleibt das für uns auch in Zukunft inakzeptabel. Die Verletzung der universellen Menschenrechte ist keine innere Angelegenheit eines einzelnen Staates, sondern ist immer zugleich auch eine Verletzung allgemeiner völkerrechtlicher Grundsätze und damit ein Problem, das auch international adressiert werden muss. Ein besonders sichtbares Beispiel ist die planmäßige Verletzung der Versammlungs- und Meinungsfreiheit in Hongkong.

Der Schutz der Religionsfreiheit weltweit ist der CDU/CSU ein besonderes Anliegen. Dabei ist für uns die vertrauensvolle Zusammenarbeit mit den Kirchen, deren humanitäre Arbeit wir fördern, eine Selbstverständlichkeit. Wir arbeiten darüber hinaus aber auch mit anderen Religionsgemeinschaften zusammen und wirken auf die Zusammenarbeit verschiedener Konfessionen hin, um interkonfessionelle Spannungen, aus denen weltweit massive Konflikte entstehen können, zu vermeiden. Wo Menschen aufgrund ihres Glaubens verfolgt werden oder staatlichen Repressalien ausgesetzt sind, setzen wir uns für sie ein – so etwa im Falle der willkürlichen Masseninhaftierungen der Uiguren in der Volksrepublik China.

### Migration ist eine der zentralen globalen Herausforderungen unserer Zeit

Das christliche Menschenbild gebietet uns, Menschen in Not zu helfen. Zugleich setzt die Integrationsfähigkeit unserer Gesellschaft der Migration Grenzen. Deswegen setzen wir uns für eine Verbesserung der Lebensbedingungen in den Herkunftsregionen sowie eine bessere Steuerung legaler Migration ein.

### Nachhaltigkeitsziele als Leitbild

Weltweit zur Überwindung der Armut, zu nachhaltiger Entwicklung und zur Stärkung der Demokratie und Sicherheit beizutragen, ist uns ein wichtiges Anliegen. Ohne Entwicklung keine Sicherheit und Stabilität, ohne Sicherheit und Stabilität keine Entwicklung. Als Christdemokraten ruht unsere Entwicklungspolitik auf drei Säulen: dem ethischen Gebot der Hilfe für die Armen und der Bewahrung der Schöpfung auch für nachfolgende Generationen, der Abwehr von Risiken für unsere Heimat sowie der Stärkung der politischen und wirtschaftlichen Verbindungen zu anderen Ländern. Die Agenda 2030 der Vereinten Nationen ist unser Leitbild für die internationale Zusammenarbeit. Wir sehen Nachhaltigkeit in ihren unterschiedlichen Ausprägungen im ökologischen, wirtschaftlichen und sozialen Bereich.

## Wo wollen wir Deutschland institutionell verbessern?

### Die Bundeswehr ist unverzichtbares Instrument deutscher Außen- und Sicherheitspolitik

Sie ist zentraler Bestandteil und Stütze des vernetzten Handelns. Die Soldatinnen und Soldaten sowie die zivilen Angehörigen der Bundeswehr leisten tagtäglich in den Einsätzen und ihren vielen anderen Aufgaben den entscheidenden Anteil dafür, dass wir in Frieden, Freiheit und Sicherheit leben können. Zugleich steht die Bundeswehr jederzeit bereit, um in Not- und Katastrophenfällen auch im Inland zu unterstützen.

Wir bekennen uns zu unserer Verantwortung für unsere Soldatinnen und Soldaten und stehen ein für ihre Anliegen. Wir bejahen soldatische Identität in der Demokratie und stehen zum Prinzip des Staatsbürgers in Uniform als Hüter von Frieden und Freiheit. Und wir treten dafür ein, den einzigartigen Dienst der Soldatinnen und Soldaten für unser Land anzuerkennen und zu würdigen.

Landesverteidigung ist heute Bündnisverteidigung. Deutschland ist gefordert, einen maßgeblichen Beitrag zur Abschreckung und Verteidigung der NATO und am Kampf gegen den Terrorismus zu schultern. Deutschland kann dies nur gemeinsam mit unseren Partnern schaffen. Deswegen muss die Bundeswehr den von ihr schon seit langem eingeschlagenen Weg der Kooperation und Integration mit den Streitkräften von Verbündeten und Partnern konsequent und innovativ weiter beschreiten. Dabei müssen wir auch die Sicherheitsbedürfnisse unserer Partner berücksichtigen und auf eine gemeinsame Strategische Kultur hinwirken.

In der NATO bedeutet dies, das Konzept der Rahmennation weiter zu stärken, und in der Europäischen Verteidigungsunion der EU über die Projekte der Ständigen Strukturierten Zusammenarbeit (SSZ; engl. PESCO) Stück für Stück eine Armee der Europäer zu formen und langfristig auf eine Europäische Armee hinzuarbeiten. Das deutsche Engagement muss in NATO und EU eine Schlüsselrolle spielen. Wir wollen sie aktiv und verantwortungsbewusst wahrnehmen.

Die Aufgabe der Auslandseinsätze bleibt weiter bestehen. Sie dienen der Wahrung deutscher Sicherheitsinteressen, der Bündnissolidarität und sind gelebte Verantwortung für Sicherheit und Frieden weltweit. Die Parlamentsbeteiligung hat sich bewährt und ist Teil des deutschen Selbstverständnisses einer Parlamentsarmee. Doch sollte es die Verständigung geben, neben der Mandatierung auf Grundlage des Art. 24 GG auch eine Mandatierung auf Grundlage Art. 87a GG vorzunehmen. In der Welt von heute wird Deutschland auch auf Distanz verteidigt.

Für ihr breites und forderndes Aufgabenprofil muss die Bundeswehr optimal ausgerüstet werden. Die Truppe muss das notwendige Material zügig erhalten. Unser Ziel bleibt die Vollausstattung. Nicht zuletzt wirkt diese sich auch auf die Attraktivität des Arbeitsgebers Bundeswehr aus. Militärische Beschaffungen wollen wir deswegen gemeinsam mit unseren europäischen Partnern effizient und kostenschonend gestalten. Außer Frage steht für uns jedoch der Erhalt einer leistungsfähigen wehrtechnischen Industrie in Deutschland als industrielle Basis unserer Sicherheitspolitik und Souveränität. Rüstungsexporte sind dabei ein gestaltendes Element deutscher Sicherheitspolitik. Deswegen setzen wir uns hierbei für einheitliche europäische Richtlinien ein.

Wir sind bereit, die für unsere Sicherheit notwendigen Finanzmittel zur Verfügung zu stellen. Die Bundeswehr braucht über Jahre stetig steigende Investitionen. Wir stehen zu unseren Zusagen an die NATO, was Finanzmittel und Fähigkeiten angeht.

### Sicherheitsarchitektur weiterentwickeln

Es ist unser Ziel, Deutschlands internationale Handlungsfähigkeit weiter zu stärken. Dafür muss die Sicherheitsarchitektur unseres Landes weiterentwickelt werden, indem die Strukturen der ressortgemeinsamen Zusammenarbeit sowie die gesamtstaatlichen und gesamtgesellschaftlichen Strategien den aktuellen Herausforderungen anpasst werden. Wir wollen darum prüfen, den Bundessicherheitsrat zu einem nationalen Sicherheitsrat weiterzuentwickeln. Durch ihn könnten alle Instrumente der Außen‑, Entwicklungs‑, Verteidigungs- und Wirtschaftspolitik besser koordiniert und ressortübergreifende Entscheidungen vereinfacht und beschleunigt werden.

## Außen- und Sicherheitspolitik für die Zukunft unseres Landes

Die CDU/CSU steht wie keine andere Partei für die Erfolgsgeschichte Deutschlands nach den Verheerungen des Zweiten Weltkriegs. Der Aufbau einer parlamentarischen Demokratie und die seinerzeit unter Konrad Adenauer gegen vehementen Widerstand des linken Spektrums durchgesetzte, unbedingte Westintegration, die erfolgreiche Aussöhnung mit Frankreich, Israel und Polen, die Wiedervereinigung Deutschlands in Frieden und Freiheit unter Helmut Kohl, zuletzt schließlich die wohlbedachte Führung Deutschlands durch die Weltfinanzkrise und durch die gegenwärtiger Covid-19-Pandemie unter Angela Merkel – in den entscheidenden Schicksalsstunden der deutschen Nachkriegsgeschichte hat die CDU/CSU mit ihrem klaren Wertekompass, der auf dem christlichen Menschenbild und den Idealen von Freiheit und Gerechtigkeit basiert, die Weichen in die richtige Richtung gestellt.

Damit auch die künftigen Generationen in unserem Land in Frieden, Freiheit und in Wohlstand leben können, müssen wir in der gegenwärtigen Welt mit ihren Umbrüchen und Herausforderungen gerade in der Außen- und Sicherheitspolitik achtsam und entschlossen handeln. Dafür steht die Fraktion der Christlich Demokratischen Union und der Christlich Sozialen Union im Deutschen Bundestag.

## Footnotes

1 Im vorliegenden Artikel wird, abweichend vom ZfAS-Standard, bei personenbezogenen Substantiven die männliche grammatikalische Form verwendet. Der Autor schließt damit Personen jeden Geschlechts gleichermaßen ein.

